# Transdermal buprenorphine patch as an adjunct to multimodal analgesia after total joint arthroplasty: a retrospective cohort study

**DOI:** 10.3389/fphar.2024.1412099

**Published:** 2024-09-20

**Authors:** Xiaoli Fang, Yueping Zhao, Yao Yao, Jianghui Qin, Yan Lin, Jin Yang, Ruijuan Xu

**Affiliations:** ^1^ China Pharmaceutical University Nanjing Drum Tower Hospital, Nanjing, China; ^2^ Department of Pharmacy, Nanjing Drum Tower Hospital, Affiliated Hospital of Medical School, Nanjing University, Nanjing, China; ^3^ School of Pharmacy, China Pharmaceutical University, Nanjing, China; ^4^ Division of Sports Medicine and Adult Reconstructive Surgery, Department of Orthopedic Surgery, Nanjing Drum Tower Hospital, Affiliated Hospital of Medical School, Nanjing University, Nanjing, China; ^5^ Department of Clinical Pharmacy, School of Basic Medicine and Clinical Pharmacy, China Pharmaceutical University, Nanjing, China; ^6^ Center of Drug Metabolism and Pharmacokinetics, China Pharmaceutical University, Nanjing, China

**Keywords:** transdermal buprenorphine patch, total joint arthroplasty, analgesia, postoperative pain, multimodal analgesia

## Abstract

**Background:**

Total hip arthroplasty or total knee arthroplasty (THA/TKA) is often associated with varying degrees of pain. In recent years, transdermal buprenorphine (TDB) patch has shown encouraging results for acute postoperative pain control in orthopedic surgery. The aim of our study was to investigate the efficacy and safety of the combination of TDB patch and nonsteroidal anti-inflammatory drugs (NSAIDs) as a multimodal analgesic regimen after THA/TKA.

**Methods:**

Patients who underwent THA and TKA between January 2022 and January 2023 were reviewed. Three postoperative analgesic regimens were selected: Group A (flurbiprofen 50 mg and tramadol 37.5 mg/acetaminophen 325 mg), Group B (flurbiprofen 50 mg and TDB 5 mg), and Group C (Parecoxib 40 mg and TDB 5 mg). The primary outcomes were the Wong-Baker face pain scale revision (FPS-R) scores and the rate of sleep disturbances. Secondary outcomes of the study included the proportion of patients with postoperative pain relief rates categorized as 0%, <50%, ≥50%, and 100%.

**Results:**

The dynamic FPS-R pain scores on day 3 after surgery in Group B were significantly lower than those in Group A for THA (*P* < 0.017). The dynamic FPS-R pain scores were lowest in Group C on day 2 and 3 after THA and TKA (*P* < 0.017). Rate of sleep disturbances was significantly lower in Group B for THA and in Group C for TKA, respectively, compared with that in Group A (*P* < 0.017). The proportion of dynamic pain relief rate ≥50% in Group C was statistically higher than that in Group A for THA (*P* < 0.017). Rate of adverse reactions among three groups for THA and TKA was not statistically different (*P* > 0.05).

**Conclusion:**

This study suggests that the combination of TDB patch and NSAIDs is safe and effective for postoperative analgesia after THA/TKA.

## 1 Introduction

Total hip arthroplasty or total knee arthroplasty (THA/TKA) is a common surgical intervention for the management of advanced hip or knee osteoarthritis, which is the third most incapacitating musculoskeletal disorder globally (Vos et al., 2012). With the aging population of the United States, THA and TKA volume is expected to increase significantly to reach an estimated 3.48 million per year by 2030 (Kurtz et al., 2007). However, these interventions are often associated with varying degrees of pain which, if left unchecked, can impede early physical therapy and rehabilitation, delay mobilization, and prolong hospital stays (Mcdonald et al., 2016).

Multimodal analgesic regimens have become the standard of care for THA/TKA, typically comprising a combination of opioids (short- and long-acting), nonsteroidal anti-inflammatory drugs (NSAIDs), gabapentinoids, acetaminophen, regional nerve blocks, and local anesthetics (Karam et al., 2021). The combination of oral or intravenous administration of opioids and NSAIDs has been demonstrated to decrease opioid usage and improve postoperative outcomes, thus being regarded as a classic multimodal analgesic regimen (Ratan et al., 2002; Buvanendran et al., 2003; Stevenson et al., 2018). However, elderly patients encounter difficulties in tolerating the serious adverse effects associated with oral or intravenous opioids, which limits their options for analgesic medications (Polomano et al., 2017).

Buprenorphine is a highly lipophilic, semi-synthetic derivative of thebaine (a morphine alkaloid) (Li et al., 2020). It is a partial agonist at μ-opioid receptors and a full antagonist at κ and δ opioid receptors. It has unique analgesic effects, no immunosuppressive effects, no cardiotoxicity, and less adverse effects of cognitive impairment. It is safe for patients suffering from renal insufficiency, and relatively safe for patients suffering from hepatic insufficiency (Likar, 2006). Additionally, buprenorphine has a lower abuse potential than other opioids because it acts primarily on the spinal cord rather than the brain (Davis, 2012).

The Transdermal buprenorphine (TDB) patch is characterized by its noninvasive nature, gradual and sustained release, and good patient adherence (Davis et al., 2018). Within 7 days of application, 15% of the patch’s drug loading capacity is continuously released and absorbed systemically to achieve a sustained effective drug concentration, resulting in a long-lasting analgesic effect. Patch application is better tolerated than oral or intravenous administration and does not require dose adjustment in the elderly (Kress, 2009; Davis et al., 2018).

Although TDB patch is routinely used for the management of chronic pain, there is also growing interest in the use for acute postoperative pain control in orthopedic surgery (Jr et al., 2021). Several randomized controlled trials have demonstrated that the preoperative use of TDB in patients undergoing hallux valgus surgery has superior analgesic efficacy compared to oral celecoxib and is comparable to intravenous flurbiprofen axetil (Xu et al., 2018). Moreover, when administered prior to fracture neck of femur and spinal surgery, TDB patch was found to be effective in reducing pain with fewer adverse effects than oral tramadol (Desai et al., 2017; Lee et al., 2017).

TDB patch has the potential to be a versatile addition to multimodal analgesic regimens in the perioperative period. However, there is a lack of studies investigating the use of TDB patch as an adjunct to postoperative multimodal analgesic regimens. In this retrospective study, we aim to compare three analgesic regimens in order to evaluate the role of TDB patch in multimodal analgesic regimens for patients undergoing THA/TKA, and we hypothesized that the addition of TDB patch would provide improved pain management. The outcome measurements included pain scores, rate of sleep disturbances and pain relief rate.

## 2 Methods

### 2.1 Study design and participants

This single-center, observational, retrospective study was conducted at Nanjing Drum Tower Hospital, Affiliated Hospital of Medical School, Nanjing University. After obtaining approval from the Ethical Committee of Nanjing Drum Tower Hospital on 1 February 2023(Number 2022-619-02), data were collected form all patients who underwent THA and TKA between January 2022 and January 2023. This study included patients aged >18 years with ASA Physical Status I–III undergoing surgery for THA/TKA. Exclusion criteria included patients lacking comprehensive records of pain scores, adverse effects, and sleep disturbances, patients using other medications and non-target analgesic regimen to relieve pain and sleep disturbances, as well as patients who were suffering from severe hepatic and renal insufficiency.

### 2.2 Therapeutic approaches

After being administered general anesthesia, all patients underwent THA/TKA procedures performed by 2 experienced surgeons. THA was performed through the direct lateral approach, while TKA was performed through the traditional anterior medial parapatellar approach. Before closing the incision, all patients were given a 30 mL peri-articular cocktail injection consisting of bupivacaine 75 mg (10 mL), compound betamethasone 5 mg: 2 mg (1 mL), epinephrine 0.3 mg (1 mL) and normal saline (100 mL).

Three postoperative analgesic regimens were selected. The selection of different analgesic regimens was determined by different medication habits among physicians and drug inventory in different time periods, and patients were divided into three groups: Group A received intravenous Flurbiprofen Axetil 50 mg (Kai Feng^®^) and oral tramadol 37.5 mg or acetaminophen 325 mg (Ji Tong An^®^) every 12 h; Group B received intravenous Flurbiprofen Axetil 50 mg (Kai Feng^®^) every 12 h and TDB patch 5 mg (Norspan^®^) 1 day before surgery; Group C received intravenous Parecoxib Sodium 40 mg (EMeiShan^®^) every 12 h and TDB patch 5 mg (Norspan^®^) 1 day before surgery. TDB patch provided continuous analgesia for 7 days.

### 2.3 Outcomes

Data were extracted from medical records, including baseline demographics, pain scores, sleep disturbances, and adverse effects.

The baseline demographics included: age, sex, body mass index, preoperative pain scores, history of analgesic medication usage, history of anxiolytic or antidepressive medication usage, postoperative drainage status, postoperative erythrocyte sedimentation rate, postoperative C-reactive protein levels and haemoglobin levels.

The Wong-Baker face pain scale revision (FPS-R) scores were used to assess patients’ level of pain. A score of 0 indicated no pain, while a score of 2 indicated slight pain, a score of 4 indicated mild pain, a score of 6 indicated moderate pain, a score of 8 indicated severe pain, and a score of 10 indicated intense pain. The primary outcomes of this retrospective study were the FPS-R pain scores within 3 days postoperatively and the rate of sleep disturbances (with or without awakening) on the first postoperative night. Secondary outcomes of the study included the proportion of patients with postoperative pain relief rates categorized as 0%, <50%, ≥50%, and 100%. The calculation of pain relief rate was as follows: (pain score on postoperative day 1 - pain score on postoperative day 3)/pain score on postoperative day 1. Patients experiencing breakthrough pain were permitted to use the rescue medication, specifically acetaminophen tablets 500 mg once. The proportion of patients using the rescue medication within the first three postoperative days were compared among the three groups. Additionally, the rates of dizziness, drowsiness, nausea, vomiting, and dry mouth within 3 days postoperatively were compared among the three analgesic regimens.

### 2.4 Study size

In the previous investigation, we observed that patients undergoing TKA experienced a higher level of pain than those undergoing THA, leading to distinct variations in pain scores (Huang and Copp, 2019). Hence, the present study was divided into THA and TKA subgroups. Based on the literature review and preliminary trial results, we determined the mean difference in pain scores to be 0.53. Using a commonly accepted standard deviation of 0.10, a two-sided alpha of 0.05, and ensuring a power of 80%, our post-hoc power analysis indicated that each group would require 68 patients (408 total).

### 2.5 Data analysis

All data collected were entered into a Microsoft Excel worksheet, and graphs were created using GraphPad Prism 9.0.0 (GraphPad Software). The Shapiro-Wilk test was used to check for normal distribution of the data. Quantitative variables are normally distributed and calculated by mean and standard deviation. Median and interquartile range were calculated for nonparametric data. Categorical variables were expressed in terms of frequency and percentages. One-way ANOVA tests were used to evaluate the significance of mean differences among the three analgesic regimens where quantitative variables met normality. Otherwise, Wilcoxon rank sum tests were performed. The Kruskal Wallis test was used for comparing median data across the three groups. Categorical data were summarized with frequencies and percentages, and the Chi-square test or Fisher’s exact test was performed. Statistical significance was evaluated at the 0.05 level. SPSS statistics version 26 (IBM Corp., Armonk, NY) was used for analysis.

## 3 Results

**FIGURE 1:**
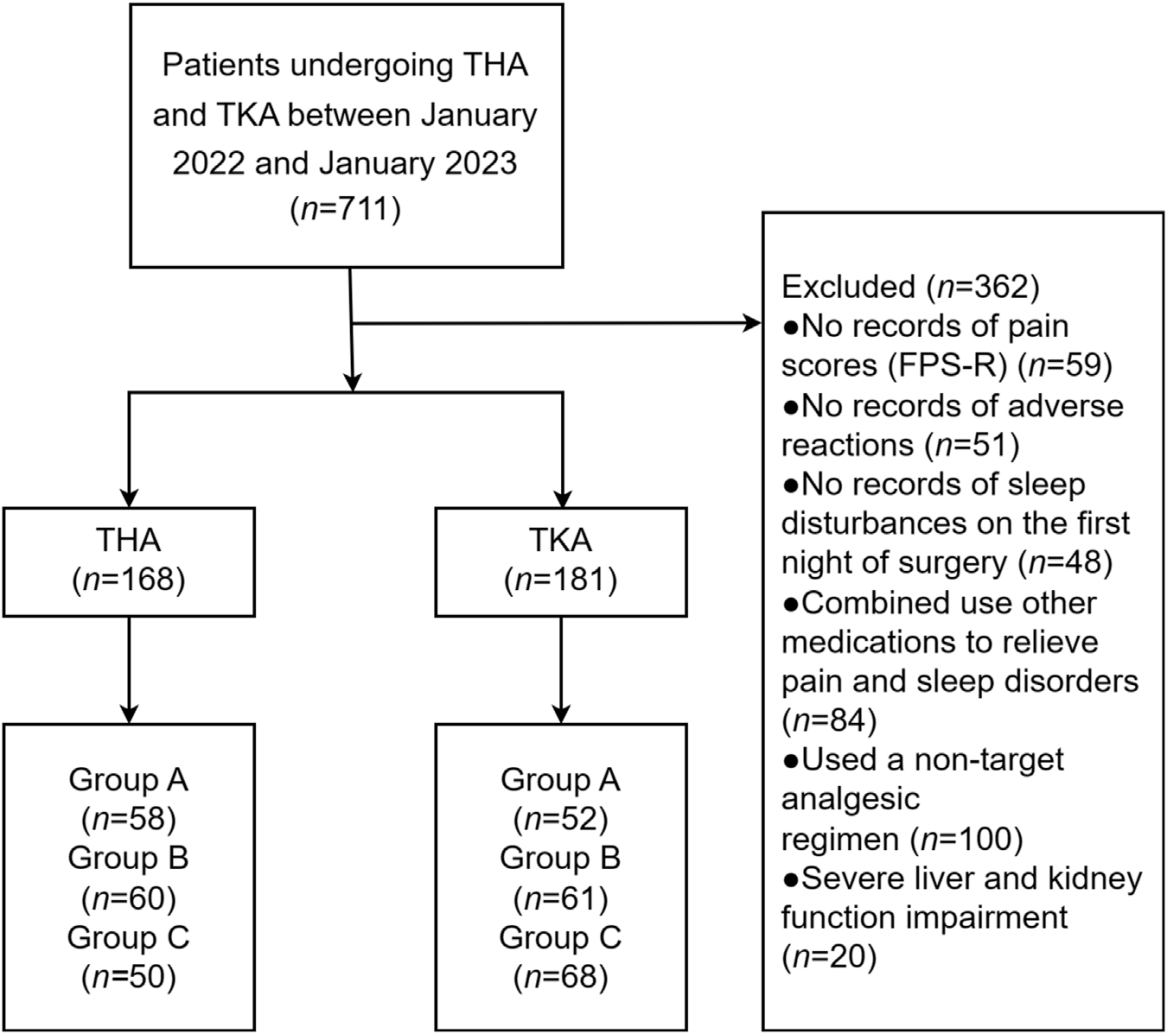
Flow diagram of patient selection.

A total of 349 patients underwent either THA (*n* = 168) or TKA (*n* = 181). Among the THA group, there were 58 patients in Group A, 60 in Group B, and 50 in Group C. In the TKA group, there were 52 patients in Group A, 61 in Group B, and 68 in Group C. Figure 1 illustrates a flow diagram depicting the study process.

The patients’ characteristics, such as age, sex, BMI, and preoperative pain scores, are listed in Table 1. There was no significant difference in terms of characteristics observed among any of the three groups for patients undergoing either THA or TKA (*P*> 0.05). For THA, the average age was 60 ± 11.4 years for Group A, 59.8 ± 12.8 years for Group B, and 60.2 ± 13.5 years for Group C. For TKA, the average age was 67.2 ± 6.6 years for Group A, 66.8 ± 8.4 years for Group B, and 65.1 ± 9.3 years for Group C.

**TABLE 1:** Baseline characteristics.

Characteristics	THA			*p*-value	TKA			*p*-value
	Group A (n = 58)	Group B (n = 60)	Group C (n = 50)		Group A (n = 52)	Group B (n = 61)	Group C (n = 68)	
Age, mean ± SD	60 ± 11.4	59.8 ± 12.8	60.2 ± 13.5	0.99	67.2 ± 6.6	66.8 ± 8.4	65.1 ± 9.3	0.32
Sex (male), n (%)	28 (48.3)	28 (46.7)	22 (44.0)	0.91	15 (28.8)	15 (24.6)	23 (33.8)	0.51
BMI, median (IQR)	24.30 (22.2–26.0)	25.2 (23.0–26.7)	24.8 (23.2–28.0)	0.43	27.5 (25.0–29.3)	28.2 (25.4–30.3)	26.9 (24.5–29.2)	0.19
Baseline static FPS-R, median (IQR)	0 (0–1.25)	0 (0–3)	0 (0–0)	0.59	0 (0–0)	0 (0–0)	0 (0–0)	0.85
Baseline dynamic FPS-R, median (IQR)	4.5 (3–6)	5 (3.3–6)	4 (3–6)	0.61	5 (5–6)	5 (4–6)	5 (4–6)	0.69
Painkillers history, n (%)	8 (13.8)	5 (8.3)	3 (6.0)	0.36	6 (11.5)	2 (3.3)	6 (8.8)	0.24
Anxiety/depression, n (%)	0	0	0	-	1 (1.9)	1 (1.6)	1 (1.5)	1
Drainage tube used^a^, n (%)	16 (27.6)	19 (31.7)	13 (26.0)	0.79	45 (86.5)	50 (82.0)	50 (73.5)	0.19
ESR^a^(mm/h), median (IQR)	20.5 (9.8–41)	17 (9.5–28.5)	17 (8–16)	0.53	16 (7.8–29.8)	14 (9–26)	13 (7–23)	0.50
CRP^a^(mg/L), median (IQR)	29 (18.4–51.3)	26.3 (15.3–49.3)	32.2 (20.5–46.2)	0.25	20.9 (15.1–44.9)	14.4 (7.3–33.2)	22.7 (11.3–40.2)	0.07
HB^a^(g/L), median (IQR)	111 (101–125)	114.5 (103.3–126.5)	114 (99.5–123.5)	0.43	118 (111–126)	117 (105–124)	122.5 (111–130)	0.17

BMI, body mass index; ESR, erythrocyte sedimentation rate; CRP, C-reactive protein; HB, hemoglobin.

^a^ postoperative.

**FIGURE 2:**
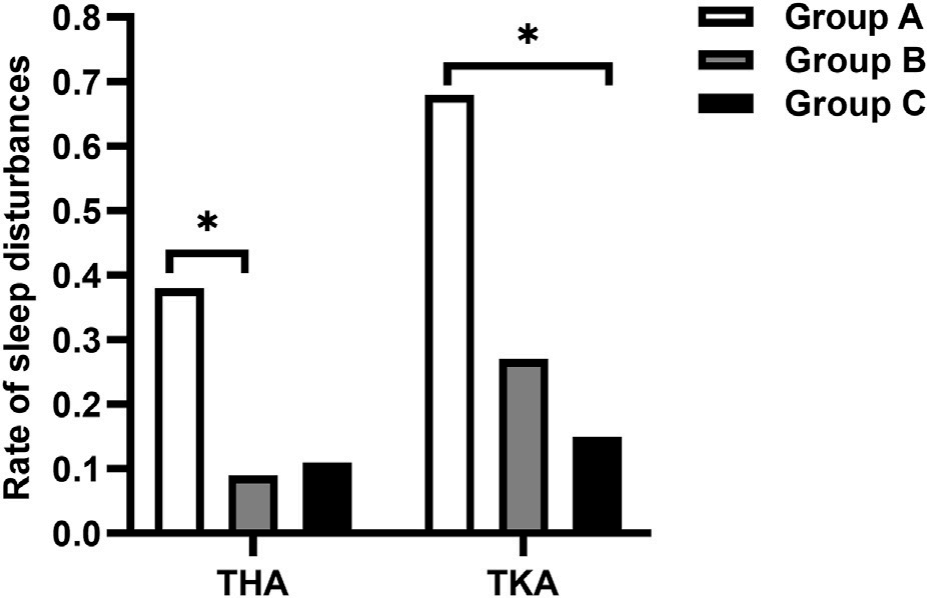
Comparison of the rate of sleep disturbances on the first postoperative night. *p-value < 0.017 vs. Group A.

No significant difference in static FSR-R pain scores was observed in any of the three groups for patients undergoing either THA or TKA. The median dynamic FSR-R pain scores were significantly lower on day 2 and 3 after surgery in the Group C compared with the Group A for THA and TKA (*p* < 0.017) (Table 2). The median dynamic FSR-R pain scores were statistically lower on day 3 after surgery in the Group B compared with the Group A for THA (*p* < 0.017) (Table 2). No statistical significance in median dynamic FPS-R pain scores was observed between Group B and Group C for THA and TKA (*P* > 0.05) (Table 2). The rate of sleep disturbances on the first postoperative night was significantly lower in Group B for THA and in Group C for TKA, respectively, compared with that in Group A (*P*< 0.017) (Figure 2).

**TABLE 2:** Comparison of static and dynamic FPS-R scores within 3 days postoperatively among the three groups.

Time (day)^a^	THA			*p*-value	TKA			*p*-value
	Group A (n = 58)	Group B (n = 60)	Group C (n = 50)		Group A (n = 52)	Group B (n = 61)	Group C (n = 68)	
Static pain								
1, median (IQR)	0 (0–2)	0 (0–0)	0 (0–0.5)	0.28	2 (0–3)	0 (0–3)	2 (0–3)	0.41
2, median (IQR)	0 (0–0)	0 (0–0)	0 (0–0)	0.34	0 (0–2)	0 (0–1.5)	0 (0–0.8)	0.27
3, median (IQR)	0 (0–0)	0 (0–0)	0 (0–0)	0.32	0 (0–1)	0 (0–0)	0 (0–0)	0.14
Dynamic pain								
1, median (IQR)	4 (4–5)	4 (4–5)	4 (4–5)	0.27	5 (4.3–6)	5 (4–5)	5 (4–6)	0.22
2, median (IQR)	4 (4–5)	3 (3–4)	3 (3–4)^*^	<0.02	5 (4–5)	4 (4–5)	4 (3.3–5)^*^	<0.017
3, median (IQR)	3 (3–4)	3 (3–3)^*^	3 (2.8–3)^*^	<0.02	4 (3–4)	3 (3–4)	3 (3–4)^*^	<0.017

^a^ postoperative.

*p-value <0.017 vs. Group A (Kruskal Wallis test).

**FIGURE 3:**
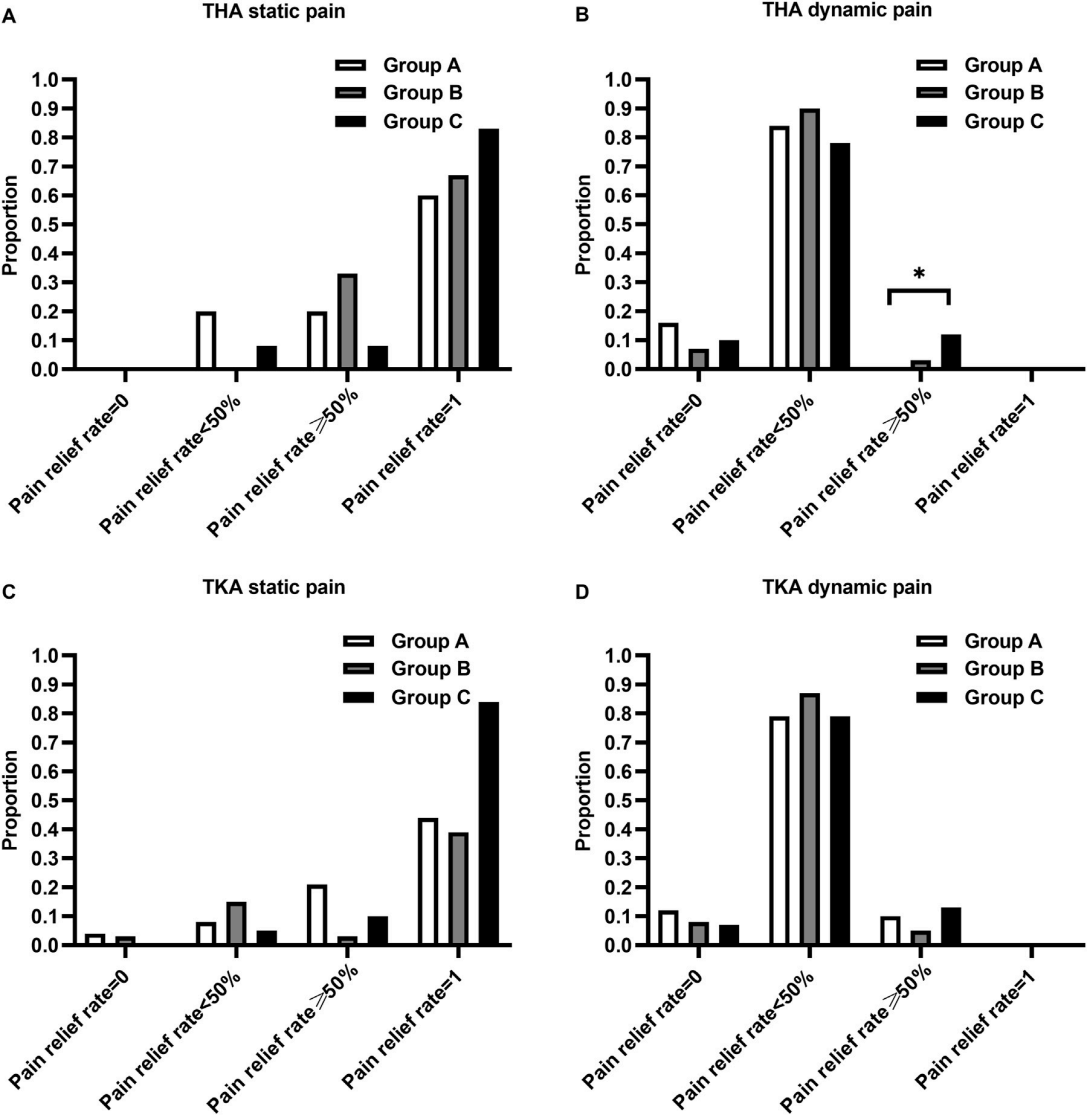
Comparison of the proportion of patients with pain relief rates categorized as 0%, <50%, ≥50%, and 100%. **(A)** Proportion of static pain relief rate in patients undergoing THA, **(B)** Proportion of dynamic pain relief rate in patients undergoing THA, **(C)** Proportion of static pain relief rate in patients undergoing TKA, **(D)** Proportion of dynamic pain relief rate in patients undergoing TKA. *p-value < 0.017 vs. Group A.

The proportion of patients with pain relief rates ≥50% in Group C was statistically higher than that in Group A for THA (*P*< 0.017) (Figure 3). The proportion of patients using the rescue medication was shown in Table 3. No significant differences of the proportion were observed in patients undergoing either TKA or THA within the first three postoperative days among the three regimen groups (*P* > 0.05). The rate of adverse reactions was shown in Table 4. No significant difference in rate of adverse reactions was observed in any of the three groups for patients undergoing either THA or TKA (*P* > 0.05).

**TABLE 3:** Comparison of the proportion of patients using rescue medications within the first 3 days postoperatively among the three groups.

Classification	Group A	Group B	Group C	*p*-value
THA				
Rate	0.02 (n = 58)	0.03 (n = 60)	0.04 (n = 50)	0.77
TKA				
Rate	0.15 (n = 52)	0.11 (n = 61)	0.10 (n = 68)	0.69

**TABLE 4:** Comparison of the rate of adverse reactions within the first 3 days postoperatively among the three groups.

Adverse reactions	THA			*p*-value	TKA			*p*-value
	Group A (n = 58)	Group B (n = 60)	Group C (n = 50)		Group A (n = 52)	Group B (n = 61)	Group C (n = 68)	
Day1^a^								
Dizziness/drowsiness, n (%)	7 (12.1)	13 (21.7)	4 (7.7)	0.11	11 (21.2)	6 (9.8)	12 (17.6)	0.24
Nausea, n (%)	2 (3.4)	4 (6.7)	1 (1.9)	0.63	8 (15.4)	2 (3.3)	8 (11.7)	0.08
Vomiting, n (%)	6 (10.3)	5 (8.3)	2 (3.8)	0.48	6 (11.5)	2 (3.3)	5 (7.3)	0.25
Dry mouth, n (%)	23 (39.7)	36 (60.0)	21 (40.4)	0.07	19 (36.5)	34 (55.7)	28 (41.2)	0.09
Day2^a^								
Dizziness/drowsiness, n (%)	7 (10.3)	3 (5.0)	4 (7.7)	0.36	7 (13.5)	2 (3.3)	3 (4.4)	0.09
Nausea, n (%)	3 (5.2)	1 (1.7)	2 (3.8)	0.59	5 (9.6)	2 (3.3)	2 (2.9)	0.26
Vomiting, n (%)	0	1 (1.7)	0	1	1 (1.9)	0	1 (1.5)	0.75
Dry mouth, n (%)	9 (15.5)	14 (23.3)	6 (11.5)	0.27	14 (26.9)	21 (34.4)	12 (17.6)	0.09
Day3^a^								
Dizziness/drowsiness, n (%)	4 (6.9)	0	1 (1.9)	0.06	3 (5.8)	0	2 (2.9)	0.23
Nausea, n (%)	3 (5.2)	0	1 (1.9)	0.16	0	0	0	—
Vomiting, n (%)	0	0	0	—	0	0	0	—
Dry mouth, n (%)	2 (3.4)	4 (6.7)	1 (1.9)	0.63	4 (7.7)	7 (11.5)	5 (7.4)	0.67

^a^ postoperative.

## 4 Discussion

The efficacy and safety of TDB patch for immediate postoperative analgesia after TKA have been confirmed through randomized controlled clinical trials, although TDB patch was generally recommended as a chronic pain killer (Londhe et al., 2020; Xu et al., 2020). However, the present study is the first to illustrate the efficacy of the combination of TDB and NSAIDs compared with the combination of tramadol and NSAIDs in the acute postoperative period of THA/TKA. The results showed that using a combination regimen of TDB patch resulted in lower dynamic pain scores and rate of sleep disturbances in the early postoperative period, which contributed to patients’ early recovery. The reduction in sleep disturbances may be attributed to continuous drug release and sustained effective drug concentration of TDB patch.

The use of TDB patch is not recommended for routine postoperative analgesia because it releases medications slowly, and reaches maximum plasma concentration gradually after approximately 24–48 h of application. In the present study, TDB patch was administered from 1 day before surgery in order to achieve the maximum plasma concentration and best analgesia effect immediately after surgery.

Preoperatively using TDB patch is also part of preemptive analgesia protocol. Preemptive analgesia is thought to reduce the incidence of postoperative hyperalgesia by reducing the memory of pain in the central nervous system and inflammation caused by cytokine and prostaglandin release (Oh, 2000). Appropriate preoperative analgesic interventions can reduce the body’s response to future nociceptive input and reduce the degree of sensitization of the central nervous system, so that the body may not feel normal pain stimuli (Woolf, 1989).

There is concern that if buprenorphine is administered prior to a full opioid agonist, the agonistic effect of buprenorphine on μ-opioid receptors may transition into antagonistic effect, thus affecting the dosage and effect of intraoperative anesthesia and postoperative opioids (Boas and Villiger, 1985; Bryson et al., 2010; Anderson et al., 2017). Several reviews concur that for mildly painful surgeries, preoperative administration of buprenorphine can be continued (Anderson et al., 2017; Kohan et al., 2021). In the case of surgeries involving moderate to severe pain, there is no clear consensus on the appropriateness of preoperative buprenorphine. Several case studies and reviews have suggested reducing the preoperative dose of buprenorphine to free up enough unoccupied μ-opioid receptors for full μ-opioid receptor agonists (Anderson et al., 2017; Jonan et al., 2018). However, a study completed by Greenwald et al. found that 71%–85% of μ-opioid receptors were still available after sublingual administration of buprenorphine up to 1 mg (0.5 mg was absorbed) (Greenwald et al., 2003). Meanwhile, the average daily absorbed dose was found to be 0.12 mg/day for a 5 mg sustained-release TDB patch over a period of 7 days (Kapil et al., 2013). These findings confirmed the potential utilization of low-dose TDB patch in conjunction with intraoperative anesthesia and postoperative opioids.

As a partial μ-opioid receptor agonist, buprenorphine exhibits a strong affinity for the receptor and demonstrates slow dissociation kinetics. Consequently, this characteristic leads to minimal downregulation of the receptor upon discontinuation and mitigates withdrawal reactions (Khanna and Pillarisetti, 2015). Moreover, Buprenorphine demonstrates a ceiling effect on respiratory inhibition with no upper limit on analgesic effect (Pergolizzi et al., 2010). As a result, the risk of potentially fatal toxic event is reduced compared with other complete opioid agonists. Adverse effects observed include dizziness, drowsiness, nausea, vomiting, and dry mouth. The incidence of these adverse effects decreased with longer hospital stays. However, no statistically significant difference was found. It is speculated that these adverse effects may be associated with post-anesthesia reactions.

The present study has limitations. Being a retrospective cohort study, the study might have inherent biases, such as selection bias. In addition, the use of TDB patch is not first-line recommended for acute postoperative analgesia, so that the enrolled sample size for the retrospective study might be limited and a larger size clinical trial with longer follow-up durations would be needed to further confirm our findings.

## 5 Conclusion

The present retrospective study illustrated that the combination of TDB patch and NSAIDs is an effective and safe multimodal analgesic regimen for postoperative pain in patients undergoing THA and TKA. TDB patch can be used as an adjunct to multimodal analgesia after THA and TKA. Further prospective studies are needed to confirm the present findings.

## Data Availability

The original contributions presented in the study are included in the article/supplementary material, further inquiries can be directed to the corresponding authors.
